# High prevalence of virulent and multidrug-resistant *Escherichia coli* as the most frequently isolated bacterium associated with bacterial pneumonia in farmed raccoon dogs

**DOI:** 10.1186/s12917-026-05773-5

**Published:** 2026-07-31

**Authors:** Hong Li, Chihuan Li, Xintong Zhu, Zhixin Zhu, Guowang Chen, Chao Ren, Siping Zhu, Tonglei Wu, Li Chen, Yonghui Li, Qiumei Shi, Zhiqiang Zhang

**Affiliations:** 1 Hebei Key Laboratory of Preventive Veterinary Medicine, Hebei Normal University of Science & Technology, Qinhuangdao, 066600 China; 2 The Second Hospital of Qinhuangdao, Qinhuangdao, 066600 China

**Keywords:** Raccoon dog, *Escherichia coli*, Hemorrhagic pneumonia, Multidrug-resistant, Pathogenicity, Multisystemic infections

## Abstract

Bacterial pneumonia is a leading cause of mortality in fur-bearing animals, representing a significant challenge for the industry. Bacterial pneumonia is caused by a variety of pathogens, making its prevention and control difficult. This study aims to identify the primary etiological agents of bacterial pneumonia in farmed raccoon dogs in eastern Hebei and southern Liaoning provinces. To this end, we collected 159 pneumonic raccoon dogs between 2022 and 2025 for bacterial isolation and identification. We isolated 109 strains of *Escherichia coli*, 37 strains of *Pseudomonas aeruginosa*, and 13 strains of *Klebsiella pneumoniae*, identifying *E. coli* as the most frequently isolated bacterium. Further investigations revealed that *E. coli* caused multisystemic infection in raccoon dogs: the bacterium was detected in the liver, spleen, kidney, and blood, with the most severe lesions observed in the lungs, suggesting potential systemic dissemination. Histopathological examination results revealed multi-organ damage consistent with bacterial infection, which correlates with the isolation of *E. coli* from affected tissues. Pathogenicity assays demonstrated that the isolated *E. coli* strains exhibited notable pathogenic potential in a mouse model, capable of reproducing typical pneumonia symptoms via intranasal inoculation. Virulence gene profiling of the isolates showed that all strains harbored at least 5 virulence genes. Among these, the virulence genes *ompR*, *phoU*, *tonB*, *fimH*, *fimC*, and *Ecs3737* exhibited high detection rates, at 94.5%, 94.5%, 93.58%, 84.4%, 77.98%, and 60.55%, respectively. Antimicrobial susceptibility testing (AST) results revealed severe multidrug resistance (MDR) among the clinical *E. coli* isolates. The isolates showed universal resistance to nalidixic acid and ampicillin, and no single antimicrobial agent was fully effective against all strains. The resistance profiles of the *E. coli* isolates varied significantly between the two geographic regions studied. Resistance gene detection showed extremely high detection rates (≥ 80%) for quinolone resistance-associated chromosomal target genes and plasmid-mediated quinolone resistance (PMQR) genes, as well as integron-associated mobile elements. The detection rates of aminoglycoside, sulfonamide, and polypeptide resistance genes ranged from 30% to 70%, while those of amphenicol, β-lactam, macrolide, and tetracycline resistance genes were between 3% and 30%. Taken together, these data indicate that *E. coli* is the most frequently isolated bacterium associated with bacterial pneumonia in sampled farmed raccoon dogs in eastern Hebei and southern Liaoning, with notable pathogenic potential in mouse models and a high prevalence of complex multidrug resistance. These findings provide critical baseline data for guiding clinical antimicrobial use and developing disease control strategies in raccoon dog farming.

**Supplementary Information:**

The online version contains supplementary material available at https://doi.org/10.1186/s12917-026-05773-5.

## Introduction

 The raccoon dog (*Nyctereutes procyonoides*) is an economically important fur animal endemic to East Asia and widely farmed across northern and northeastern China [1, 2]. In China, the Ussuri raccoon dog is the dominant commercial breeding breed contributing substantially to local livestock economy, with eastern Hebei and southern Liaoning developing into key intensive farming zones owing to suitable climate and geography [3]. In China, the Ussuri raccoon dog is the primary breed for commercial farming and contributes greatly to the regional livestock economy. However, intensive rearing has facilitated frequent outbreaks of bacterial pneumonia, an acute, highly contagious disease with high mortality that mainly affects young raccoon dogs from September to November and causes massive economic losses to the fur farming industry.

In recent years, with the intensification of farming density and escalating environmental stress, bacterial pneumonia has become one of the primary diseases threatening the health of farmed raccoon dogs and the economic benefits of the industry [4]. Characterized by acute onset, rapid transmission, and high mortality, bacterial pneumonia exerts particularly severe impacts on rapidly growing juvenile raccoon dogs from September to November, frequently causing acute mortality in farmed populations and substantial economic losses [5].

The etiology of bacterial pneumonia in fur-bearing animals is diverse, with *Pseudomonas aeruginosa*, *Escherichia coli*, and *Klebsiella pneumoniae* all reported as dominant causative pathogens [6]. Although multiple bacterial species have been implicated in pneumonia of other fur animals, there remains a critical and unresolved knowledge gap regarding the definite primary etiological agent responsible for pneumonia in farmed raccoon dogs. To date, no consensus has been reached on the predominant pathogen, and relevant systematic epidemiological evidence is still lacking, making this issue highly controversial among relevant studies [4].

*Escherichia coli* (*E. coli*), as a classic opportunistic pathogen, has been previously implicated in pneumonia outbreaks in farmed foxes and minks, yet critical gaps remain in the prevention and control of this pathogen in raccoon dog farming [7, 8]. First, *E. coli* harbors an extensive diversity of serotypes, and the dominant circulating serotypes vary markedly across geographical regions. This poses a major challenge for vaccine development, as many currently available vaccines show limited protective efficacy and fail to provide comprehensive coverage against prevalent epidemic serotypes [9, 10].

Second, excessive and inappropriate use of antibiotics in clinical practice has led to a severe antimicrobial resistance (AMR) crisis, with the frequent emergence of multidrug-resistant (MDR) strains. The resistance rate of some *E. coli* isolates to commonly used antimicrobials, including β-lactams and fluoroquinolones, has exceeded 50%, drastically compromising the effectiveness of clinical treatment [11]. In addition, the relatively inadequate biosecurity protocols in fur-bearing animal farming facilities have resulted in recurrent disease outbreaks, creating a challenging situation for disease prevention and control. Therefore, systematically analyzing the pathogenic characteristics of *E. coli* and clarifying its serotype distribution, antibiotic resistance profiles, and virulence gene composition are crucial for developing scientific prevention and control strategies, as well as targeted vaccines and treatment protocols [4].

Accordingly, this study focused on the major raccoon dog farming-intensive regions of eastern Hebei and southern Liaoning. From 2023 to 2025, pneumonia cases were systematically sampled and subjected to etiological identification, antimicrobial resistance detection, and pathogenicity evaluation of the dominant isolates. This study specifically aimed to characterize bacterial isolates recovered from pneumonic raccoon dogs and identify the most prevalent bacterial taxa associated with raccoon dog pneumonia within major intensive farming zones of eastern Hebei and southern Liaoning.

## Materials and methods

### Sample collection and bacterial isolation

Between 2022 and 2025, 159 tissue samples (including lung, liver, spleen, and other organ tissues) were collected from 159 individual raccoon dogs that died of suspected bacterial pneumonia at 98 commercial farms in eastern Hebei and southern Liaoning provinces. Farms were selected based on convenience sampling from regions with high raccoon dog farming density and pneumonia outbreaks. Only animals showing typical clinical signs of pneumonia (coughing, dyspnea, nasal discharge) and gross pathological lesions at necropsy were included in the study. Each tissue sample was approximately 1–2 g, collected aseptically using disposable sterile instruments to avoid cross-contamination. All samples were then homogenized in sterile phosphate-buffered saline (PBS), inoculated onto sheep blood agar (SBA) plates, and incubated aerobically for 16–24 h at 37 °C in a 5% CO₂-enriched atmosphere. Additional organ samples (liver, spleen, kidney, etc.) were collected from a subset of animals with severe systemic lesions for further investigation. Purified single colonies were subjected to Gram staining, biochemical identification, and 16 S rRNA gene sequencing. Biochemical identification included tests for indole production, methyl red, Voges-Proskauer, citrate utilization, lactose fermentation, glucose fermentation, urease activity, and oxidase production using the API 20E system (bioMérieux, France) according to the manufacturer’s instructions. The 16 S rRNA gene was amplified using the universal primer pair 27 F (5′-AGAGTTTGATCMTGGCTCAG-3′) and 1492R (5′-TACGGYTACCTTGTTACGACTT-3′). PCR amplicons were purified and subsequently subjected to bidirectional sequencing. The obtained sequences were queried against the NCBI GenBank database via BLAST analysis. Sequences sharing ≥ 97% sequence identity were classified as belonging to the same species.

Lung, liver, spleen, kidney, thymus, and other relevant organs were selected from diseased raccoon dogs, fixed in 4% paraformaldehyde (PFA), and routinely processed for hematoxylin and eosin (H&E) staining.

### Serotype identification

Serotype identification of *E. coli* isolates was performed using the slide agglutination test. Monovalent *E. coli* O-antigen typing sera were purchased from the China Institute of Veterinary Drug Control (IVDC, Beijing, China). Briefly, an equal volume of the bacterial suspension to be tested and O-antigen-specific serum were dropped onto a clean glass slide, with a mixture of bacterial suspension and sterile normal saline set as the negative control. A reaction was considered positive if specific agglutination occurred within 1 min [12, 13].

### Pathogenicity assay of isolates in mice

Five *E. coli* isolates were randomly selected for in vivo infection assays, representing the most prevalent O-serotypes (O8, O25, O78) and both geographic regions, the details of the selected isolates are illustrated in Supplementary Table 1. A total of 60 healthy 6-week-old Kunming mice were purchased from SPF (Beijing) Biotechnology Co., Ltd. The mice were randomly divided into 6 groups with 10 mice per group. *Escherichia coli* isolates were cultured in Luria-Bertani (LB) broth to the mid-logarithmic growth phase, and the bacterial culture was adjusted to a concentration of 1 × 10^7^ CFU/mL with sterile phosphate-buffered saline (PBS). The inoculation was performed under light isoflurane anesthesia using a gentle technique to minimize stress. Mice in the infection groups were inoculated intranasally with 0.2 mL of the bacterial suspension per mouse, while mice in the control group were inoculated with an equal volume of sterile PBS. All mice were observed continuously for 7 days, and clinical signs and mortality were recorded. At 7 days post-infection, mice were euthanized to observe gross pathological changes in the lungs. One mouse was randomly selected from the PBS control group, and lung tissues from all euthanized mice were fixed in 4% PFA and processed for H&E staining. Euthanasia was performed by intraperitoneal injection of pharmaceutical-grade pentobarbital sodium (150 mg/kg) when mice exhibited severe clinical signs of distress, such as weight loss exceeding 20%, lethargy, or respiratory distress, consistent with AVMA guidelines.

In addition, one *E. coli* isolate was randomly selected and cultured to the mid-logarithmic growth phase. The bacterial suspension was adjusted to serial concentrations, and mice were inoculated intranasally with 1 × 10⁸, 1 × 10⁷, 1 × 10⁶, 1 × 10⁵, 1 × 10⁴, and 1 × 10³ CFU per mouse, respectively. The control group was inoculated with an equal volume of PBS. All mice were observed continuously for 7 days, and clinical symptoms and mortality were recorded. The LD₅₀ was determined using the Reed-Muench method.

### Virulence gene detection

Bacterial genomic DNA was extracted using a commercial bacterial genomic DNA extraction kit (Aidlab Biotech, Beijing, China) following the manufacturer’s instructions. A total of 18 virulence genes associated with *E. coli* pathogenicity were detected by PCR. The selected genes were chosen based on their established roles in adhesion, invasion, iron acquisition, and regulation of virulence in previous studies of respiratory and systemic infections, including iron uptake-related genes (*tonB*, *fyuA*, *ireA*, *iutA*, *iucD*), bacterial adhesion and colonization-related genes (*fimH*, *fimC*, *eaeA*, *vat*, *sfa*), immune evasion-related genes (*ompR*, *phoU*, *ompT*, *Iss*, *hlyF*), toxin-related genes (*cnf*, *hlyD*), and the ETT2 pathogenicity island-related gene *Ecs3737*. These virulence genes were selected based on their well-documented roles in *E. coli* pathogenicity in prior studies. The primer sequences used for detection were derived from previously reported literature [9], and detailed information is presented in Table 1. All primers were synthesized by Sangon Biotech (Shanghai) Co., Ltd.

**Table 1:** Primers used for virulence gene detection

Primer	Nucleotide Sequence (5’–3’)	Amplicon Size (bp)	Source
tonB-F	AGCCGATTTCTGTCACGA	360	This study
tonB-R	GATACTGCGGCTGATTAC		
fyuA-F	TGATTAACCCCGCGACGGGAA	787	This study
fyuA-R	CGCAGTAGGCACGATGTTGTA		
ireA-F	GATGACTCAGCCACGGGTAA	254	This study
ireA-R	CCAGGACTCACCTCACGAAT		
iutA-F	GGCTGGACATCATGGGAACTGG	302	This study
iutA-R	CGTCGGGAACGGGTAGAATCG		
iucD-F	ACAAAAAGTTCTATCGCTTCC	692	This study
iucD-R	CCTGATCCAGATGATGCTC		
fimH-F	TCGAGAACGGATAAGCCGTGG	508	This study
fimH-R	GCAGTCACCTGCCCTCCGGTA		
fimC-F	GGGTAGAAAATGCCGATGGTG	497	This study
fimC-R	CGTCATTTTGGGGGTAAGTGC		
eaeA-F	TAACGGCTATTTCCGCATGA	552	This study
eaeA-R	TCCCAGACGATACGATCCAG		
vat-F	GTTGGTGGCAACAATCCT	549	This study
vat-R	ATACAGCTGAATGCCTTC		
sfa-F	GGCAGAGGGCCGGCAACAGGC	594	This study
sfa-R	CCCGTAACGCGCCAGCATCTC		
ompR-F	AAGGCTTCCAGGTTCGAA	441	This study
ompR-R	TGACCAGTGCCTTCAGTA		
phoU-F	GAGCAGCAGCTTTCTGAT	610	This study
phoU-R	CTGCCAGCAGTTTATCGA		
ompT-F	TCATCCCGGAAGCCTCCCTCACTACTAT	496	This study
ompT-R	TAGCGTTTGCTGCACTGGCTTCTGATAC		
Iss-F	CAGCAACCCGAACCACTTGATG	323	Zhang et al., 2025 [9]
Iss-R	AGCATTGCCAGAGCGGCAGAA		
hlyF-F	GGCCACAGTCGTTTAGGGTGCTTACC	450	Zhang et al., 2025 [9]
hlyF-R	GGCGGTTTAGGCATTCCGATACTCAG		
cnf-F	ATCTTATACTGGATGGGATCATCTTGG	1105	This study
cnf-R	GCAGAACGACGTTCTTCATAAGTATC		
hlyD-F	CTCCGGTACGTGAAAAGGAC	904	This study
hlyD-R	GCCCTGATTACTGAAGCCTG		
Ecs3737-F	TACTAATGCCATATAGCCCCATAA	445	Zhang et al., 2025 [9]
Ecs3737-R	CTACGCTTTTAACAAACGATTGAT		

### Antimicrobial susceptibility testing

Antibiotic susceptibility testing (AST) of the 109 *E. coli* isolates was performed using the Kirby-Bauer (K-B) disk diffusion method in accordance with the Clinical and Laboratory Standards Institute (CLSI) 2022 guidelines (M100-S32). A total of 23 antimicrobial agents were selected for antimicrobial susceptibility testing (AST). The disk contents (µg per disk) were as follows: imipenem (10 µg), nitrofurantoin (300 µg), nalidixic acid (30 µg), trimethoprim (5 µg), cefoxitin (30 µg), cefotaxime (30 µg), ciprofloxacin (5 µg), sulfamethoxazole (250 µg), azithromycin (15 µg), gentamicin (10 µg), ampicillin (10 µg), meropenem (10 µg), tobramycin (10 µg), netilmicin (30 µg), levofloxacin (5 µg), minocycline (30 µg), amikacin (30 µg), streptomycin (10 µg), augmentin (amoxicillin 20 µg/clavulanic acid 10 µg), norfloxacin (10 µg), ofloxacin (5 µg), tetracycline (30 µg), and cefazolin (30 µg). Briefly, mid-log phase bacterial cultures were adjusted to a 0.5 McFarland standard and inoculated onto 4 mm-deep Mueller-Hinton (MH) agar plates (Qingdao Haibo Biotechnology Co., Ltd., Qingdao, China). Susceptibility disks (Hangzhou Microbial Reagent Co., Ltd., Hangzhou, China) were placed aseptically right after inoculation with proper disk spacing, followed by aerobic incubation at 37 °C for 16–18 h. Zone diameters were measured to the nearest millimeter using a digital caliper.

AST results were interpreted as Susceptible (S), Intermediate (I), or Resistant (R) according to CLSI M100 standard for *Enterobacterales*. *E. coli* ATCC 25,922 was used as the quality control strain for all assays. Multidrug-resistant (MDR) strains were defined as isolates resistant to three or more classes of antimicrobial agents.

AST results were interpreted according to CLSI M100-S32 (2022) breakpoints for Enterobacterales. Multidrug resistance (MDR) was defined as resistance to three or more different antimicrobial classes, as described by Magiorakos [14].

Considering the high degradability of imipenem and meropenem, we further determined the isolates’ resistance to the two antibiotics using the MIC assay.

The MIC assay was performed using the broth microdilution method following CLSI M100-S32 standards. All isolates preliminarily classified as imipenem or meropenem non-susceptible by disk diffusion were included for confirmation. Detailed isolate identifiers (consistent with the main dataset) and corresponding MIC values are provided in Supplementary Table S2. Of note, carbapenemase-encoding genes were not screened in this study, and the molecular mechanism underlying the observed carbapenem non-susceptibility phenotype requires further investigation.

### Resistance gene detection

A total of 25 resistance genes were detected via PCR, including β-lactam resistance genes (*bla*_*TEM*_, *bla*_*SHV*_, *bla*_*OXA*_, *bla*_*CTX−M*_, *bla*_*CMY*_), quinolone resistance-associated chromosomal target genes (*gyrA*, *gyrB*, *parC*) and plasmid-mediated quinolone resistance genes (*oqxB*, *qnrS*), aminoglycoside resistance genes (*aadA1*, *aadB*, *aac(3’)-iia*), macrolide resistance gene *mph(A)*, florfenicol resistance genes (*floR*, *fexA*, *pexA*), tetracycline resistance genes (*tetA*, *tetB*, *tetC*), sulfonamide resistance genes (*sul1*, *sul2*), polymyxin/colistin resistance gene *mcr-1*, and integron-associated genes (*intl1*, *intl2*). PCR was performed in a 25 µL reaction volume containing 12.5 µL of 2×Taq PCR MasterMix (Tiangen Biotech), 1 µL of each primer (10 µM), 2 µL of template DNA (50–100 ng/µL), and 8.5 µL of nuclease-free water. The cycling conditions were as follows: initial denaturation at 94 °C for 5 min; 30 cycles of denaturation at 94 °C for 30 s, annealing at 52–58 °C (optimized for each primer set) for 30 s, and extension at 72 °C for 1 min; and a final extension at 72 °C for 10 min. PCR products were analyzed by electrophoresis on 1.5% agarose gels stained with ethidium bromide and visualized under UV light. *E. coli* ATCC 25,922 was used as a negative control, and previously confirmed positive isolates were used as positive controls for each gene. All primers were synthesized by Sangon Biotech (Shanghai, China), and their sequences and amplicon sizes are listed in Table 2 [9, 15].

**Table 2:** Primer sequences used for resistance gene detection

Primer	Nucleotide Sequence (5’–3’)	Amplicon Size (bp)	Source
bla_TEM_-F	GCATCTTACGGATGGCATGA	99	This study
bla_TEM_-R	TTTCTTGGCTTTTATGCTTG		
bla_SHV_-F	TGACGGTCGGCGAACTCT	795	This study
bla_SHV_-R	ATTGGCGGCGCTGTTATC		
bla_OXA_-F	TTTTCTGTTGTTTGGGTTTC	121	This study
bla_OXA_-R	ATTCATTACGTATAGATGCC		
bla_CTX−M_-F	ATGGTGACAAAGAGAGTGCAAC	875	Gao et al., 2018 [15]
bla_CTX−M_-R	TTACAGCCCTTCGGCGATGATTC		
bla_CMY_-F	ATGATGAAAAAATCGTTATG	1146	Gao et al., 2018 [15]
bla_CMY_-R	TTATTGCAGCTTTTCAAGAATG		
gyrA-F	GGTGACGTAATCGGTAAATA	810	This study
gyrA-R	ACCATGGTGCAATGCCACCA		
gyrB-F	GGACAAAGAAGGCTACAGCA	879	This study
gyrB-R	CGTCGCGTTGTACTCAGATA		
parC-F	CTGGGTAAATACCATCCGCAC	987	This study
parC-R	CGGTTCATCTTCATTACGAA		
oqxB-F	TTCTCCCCCGGCGGGAAGTAC	512	This study
oqxB-R	CTCGGCCATTTTGGCGCGTA		
qnrS-F	GCAAGTTCATTGAACAGGGT	619	This study
qnrS-R	TCTAAACCGTCGAGTTCGGCG		
aac (3’)-iia-F	GGCGACTTCACCGTTTCT	412	This study
aac (3’)-iia-R	GGACCGATCACCCTACGAG		
aadA1-F	ACTATCAGAGGTAGTTGGCG	752	This study
aadA1-R	ATTTGCCGACTACCTTGGTG		
aadB-F	GAGGAGTTGGACTATGGATT	208	This study
aadB-R	CTTCATCGGCATAGTAAAA		
mph(A)-F	ATGACCGTAGTCACGACCG	906	Zhang et al., 2025 [9]
mph(A)-R	TCATTCCGCTGCGGCGAGC		
floR-F	TCCTGAACACGACGCCCGCTAT	960	This study
floR-R	TCACCGCCAATGTCCCGACGAT		
fexA-F	CTCTTCTGGACAGGCTGGAA	332	This study
fexA-R	CCAGTTCCTGCTCCAAGGTA		
pexA-F	CTTCCAGTTGAGAAGCGAGC	320	This study
pexA-R	AGAAGCATACCCGTGAACATG		
tetA-F	TACGGGAGTTTGTTGGA	525	This study
tetA-R	GTATGGAGGATGTGGTTTC		
tetB-F	TTGGTTAGGGGCAAGTTTTG	659	This study
tetB-R	GTAATGGGCCAATAACACCG		
tetC-F	ACTTGGAGCCACTATCGAC	427	This study
tetC-R	CTACAATCCATGCCAACCC		
sul1-F	AGGCTGGTGGTTATGCACTC	267	This study
sul1-R	CACCGAGACCAATAGCGGAA		
sul2-F	TCATCTGCCAAACTCGTCGT	266	This study
sul2-R	GACAGAAGCACCGGCAAATC		
mcr-1-F	CGGTCAGTCCGTTTGTTC	309	This study
mcr-1-R	CTTGGTCGGTCTGTAGGG		
intl1-F	GGGTCAAGGATCTGGATTTCG	475	Zhang et al., 2025 [9]
intl1-R	ACATGCGTGTAAATCATCGTCG		
intl2-F	CACGGATATGCGACAAAAAGGT	750	Zhang et al., 2025 [9]
intl2-R	GTAGCAAACGAGTGACGAAATG		

## Results

### *Escherichia coli* represents the most frequently isolated bacterial taxon from pneumonic raccoon dog samples

**Fig. 1:**
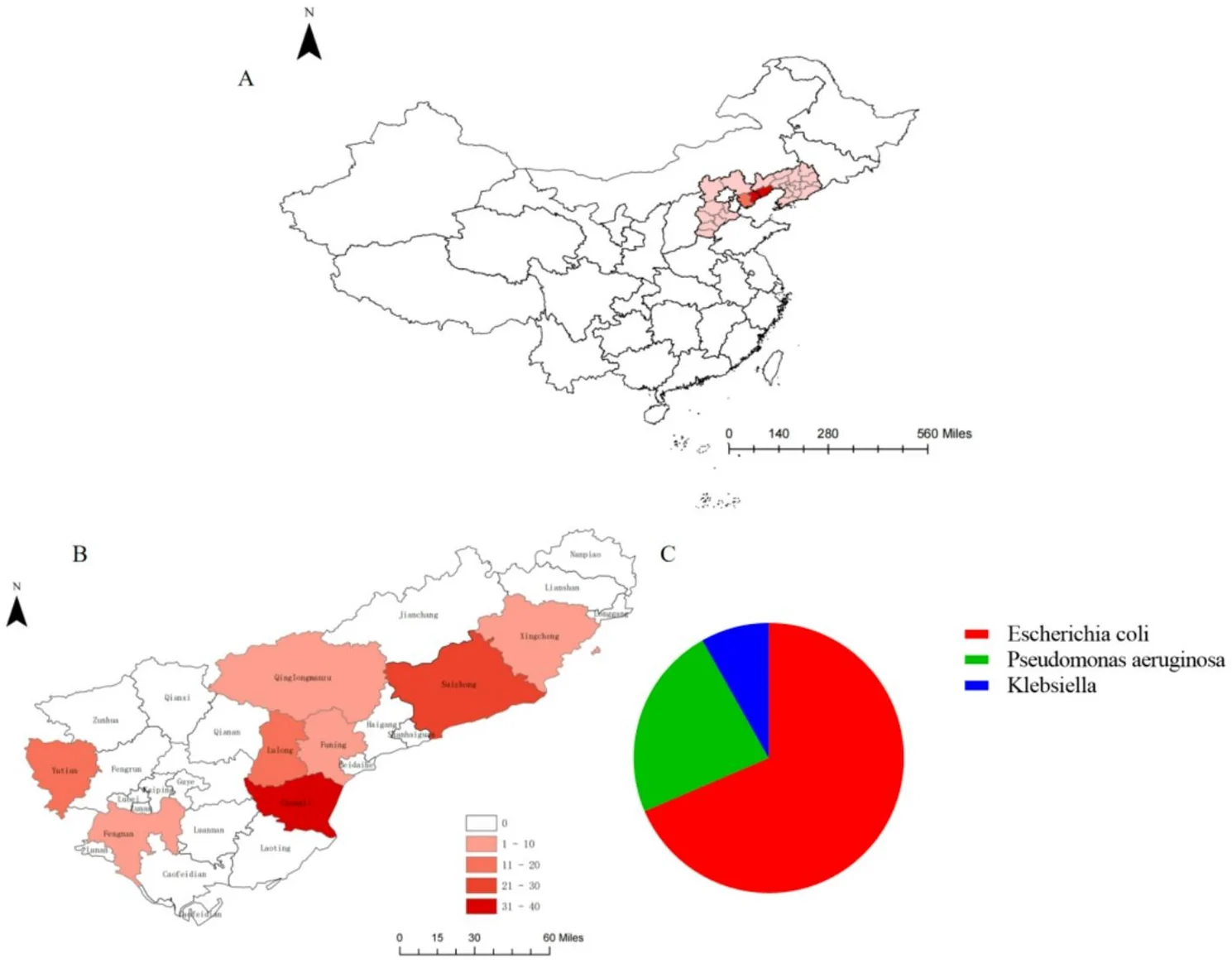
Sampling location distribution and composition of bacterial isolates recovered from pneumonic raccoon dogs in eastern Hebei and southern Liaoning. **A** Schematic map of China highlighting the core raccoon dog intensive farming regions (eastern Hebei and southern Liaoning) selected for sample collection in this study. **B** Detailed regional distribution map showing the quantity of *E. coli* isolates recovered from each sampling county; gradient color blocks correspond to isolate numbers ranging from 1 to 10, 11–20, 21–30 and 31–40. **C** Pie chart illustrating the proportional composition of three dominant bacterial pathogens isolated from raccoon dog pneumonia samples: red = *Escherichia coli*, green = *Pseudomonas aeruginosa*, blue = *Klebsiella pneumoniae*

In this study, a total of 159 lung tissue samples were collected from 159 individual raccoon dogs with clinical pneumonia at 98 affected farms in the intensive raccoon dog farming areas of eastern Hebei and southern Liaoning provinces. Sampling was performed using a convenience sampling design targeting farms with reported pneumonia outbreaks. Each sample represents a distinct outbreak of bacterial pneumonia. The geographic distribution of sampling sites is shown in Fig. 1A.

Following bacterial isolation and identification using biochemical tests and 16 S rRNA gene sequencing, a total of 159 bacterial isolates were recovered from the 159 samples. These included 109 *E. coli* isolates (68.6%), 37 *P. aeruginosa* isolates (23.3%), and 13 *K. pneumoniae* isolates (8.2%). Descriptive distribution analysis showed that *E. coli* was the most frequently isolated bacterium among the examined samples.

Geographically, 77 *E. coli* isolates (70.6%) were recovered from Hebei Province, and 32 isolates (29.4%) were from Liaoning Province (Fig. 1B). Figure 1B clearly indicates the number of samples collected per region to allow assessment of sampling intensity.

### *E. coli* can be isolated from multiple organs of pneumonic raccoon dogs, indicating potential systemic dissemination

The primary clinical signs observed in affected animals (*n* = 159) included coughing (100%), dyspnea (92.5%), nasal discharge (87.4%), and lethargy (79.2%). Based on farm records and field observations, the disease progression was rapid, with 78.6% (902/1148) of affected animals dying within 48 h of the onset of clinical signs.

**Fig. 2:**
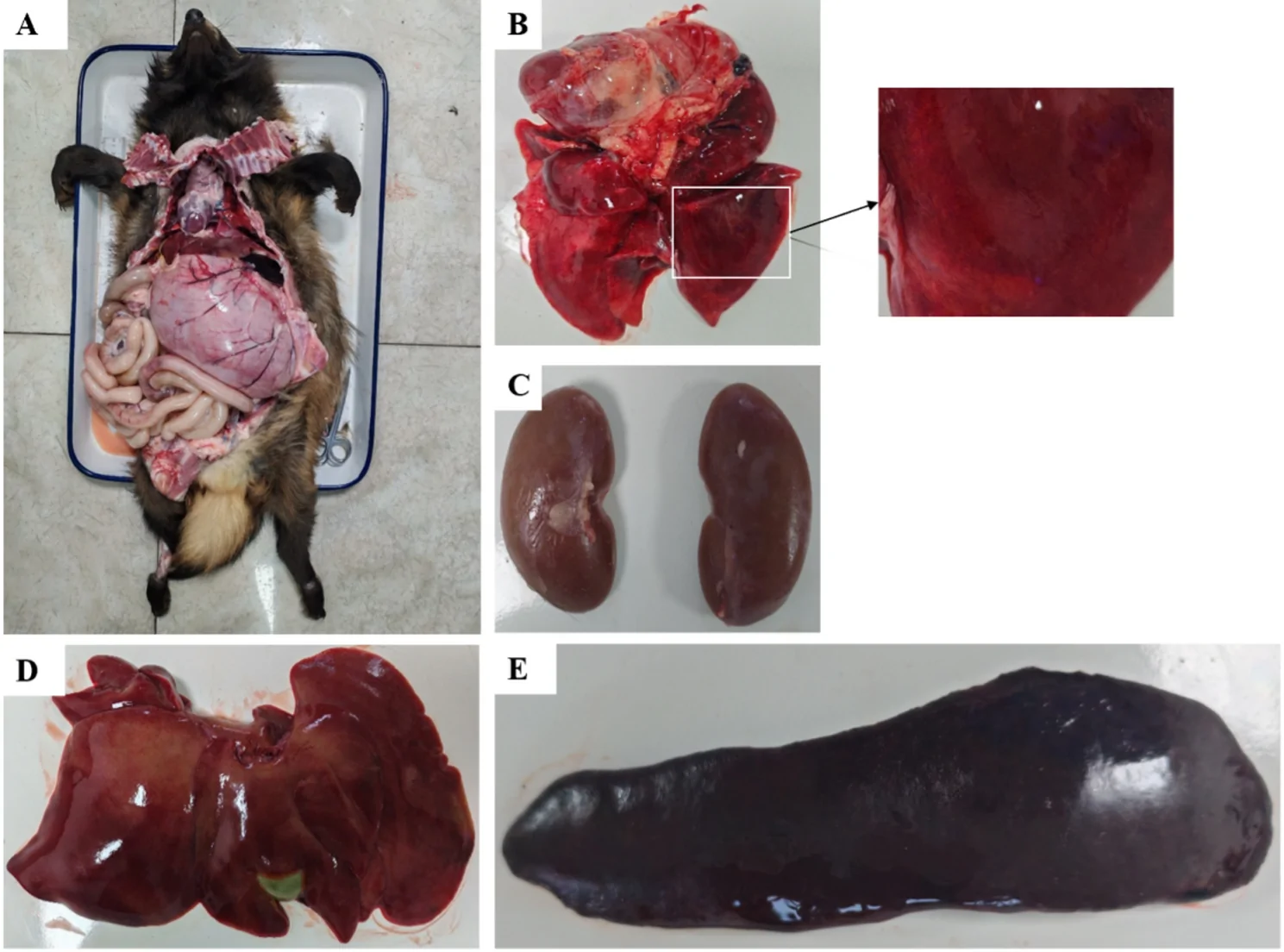
Organs and tissues of raccoon dogs infected with *E. coli ***A**. Raccoon dog infected with *E. coli ***B**. Heart and lungs of raccoon dog infected with *E. coli ***C**. Kidneys of raccoon dog infected with *E. coli ***D**. Liver of raccoon dog infected with **E**. coli E. Spleen of raccoon dog infected with *E. coli*

At necropsy, all 159 animals showed severe pulmonary lesions, including hemorrhage (100%). Systemic lesions were observed in 32 *E. coli* positive animals (29.4%), including hemorrhage in the liver (18.9%), spleen (15.1%), and kidneys (12.6%) (Fig. 2). *E. coli* was isolated from multiple organs, including liver, spleen, kidney, blood, and brain in all 32 animals, which is consistent with possible systemic bacterial dissemination.

**Fig. 3:**
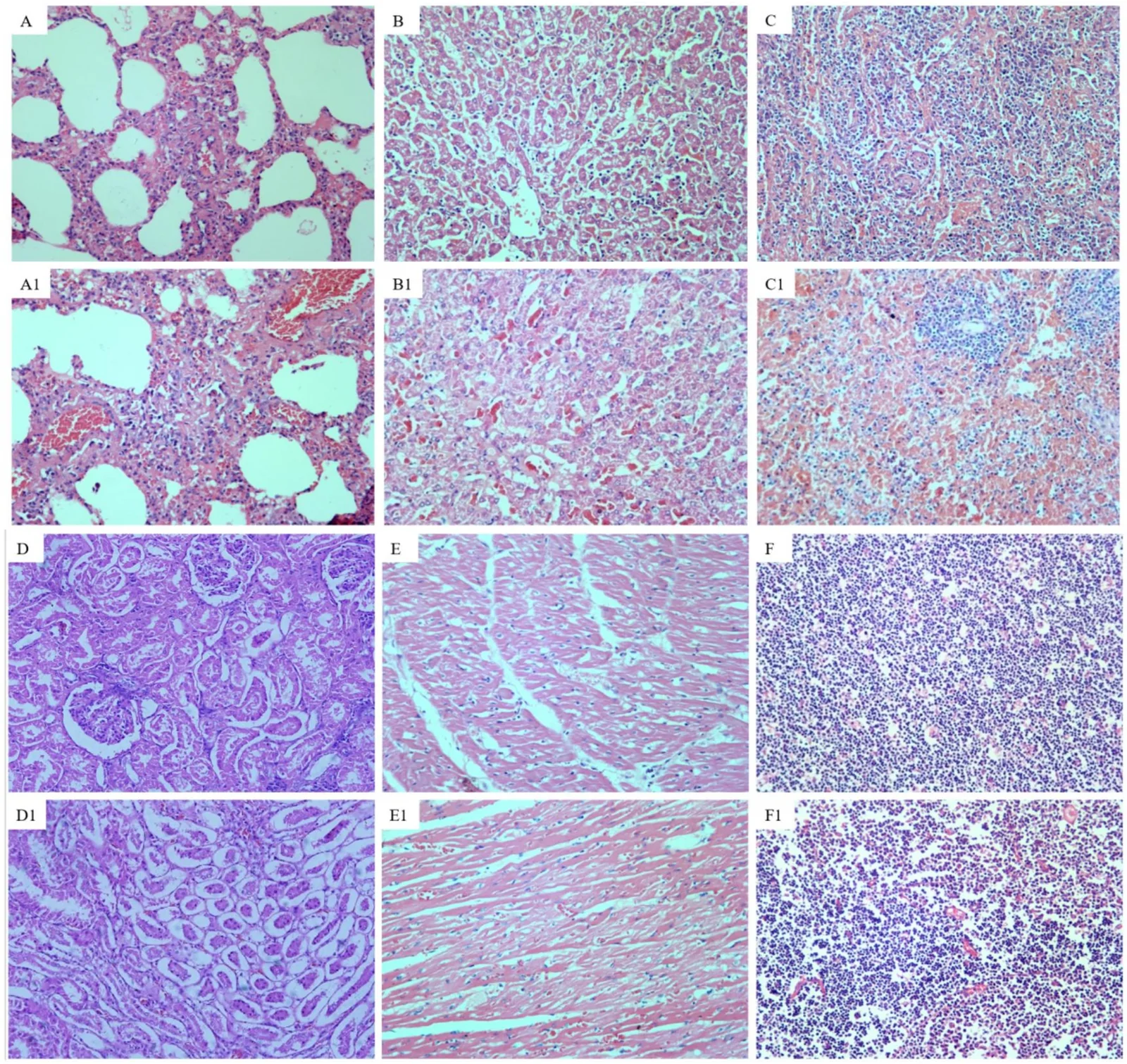
Histopathological changes in the organs and tissues of raccoon dogs infected with *E. coli*. A-F Healthy raccoon dog organs, A1-F1 Organs after *E. coli* infection. (A, A1) Lungs, (B, B1) Liver, (C, C1) Spleen, (D, D1) Kidneys, (E, E1) Heart, (F, F1) Thymus. Tissues were observed under a 200× magnification after hematoxylin-eosin staining

Hematoxylin and eosin (H&E) staining showed dilation and congestion of pulmonary blood vessels, inflammatory cell infiltration in the pulmonary interstitium and alveolar spaces, and alveolar wall hemorrhage (Fig. 3A1). The hepatic cord structure was disorganized, with congestion in the hepatic sinusoids (Fig. 3B1). A large number of inflammatory cells were distributed in the splenic parenchyma, the structural boundary between normal white pulp and red pulp was lost, erythrocyte aggregation was observed in local areas, and the splenic tissue structure was disorganized (Fig. 3C1). Renal tubular epithelial cells showed vacuolar degeneration, accompanied by inflammatory cell infiltration, congestion and hemorrhage in the renal interstitium (Fig. 3D1). The myocardial interstitium was congested with a small amount of erythrocyte exudation (Fig. 3E1). Hemorrhage and massive inflammatory cell infiltration were observed in the thymus tissue (Fig. 3F1). These histopathological changes are consistent with bacterial infection, and the isolation of *E. coli* from these tissues supports a potential association with the observed lesions, but cannot confirm a definitive causal role of *E. coli* in multi-organ damage.

### O8, O25, and O78 are the most detected serotypes of *E. coli* isolates

**Fig. 4:**
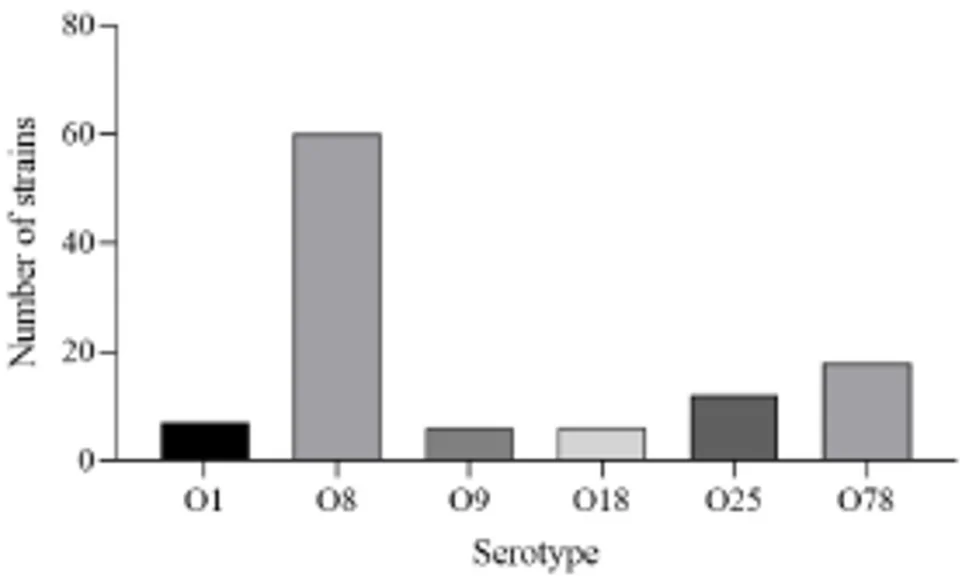
Distribution of serotypes among 109 *E. coli* strains isolated from farmed raccoon dogs with pneumonia. The serotypes of the isolates were determined, with O8 being the dominant serotype, followed by O78 and O25. Other serotypes, including O1, O9, and O18, were detected at lower frequencies

The results of O-serotyping showed that A total of 6 O-serotypes were detected among the 109 *E. coli* isolates: O1 (7/109, 6.42%), O8 (60/109, 55.05%), O9 (6/109, 5.5%), O18 (6/109, 5.5%), O25 (12/109, 11.01%), and O78 (18/109, 16.51%). O8, O25, and O78 were the most frequently detected serotypes among isolates (Fig. 4).

### *E. coli* isolates induce pneumonia-like lesions and exhibit pathogenic potential in a mouse infection model

**Fig. 5:**
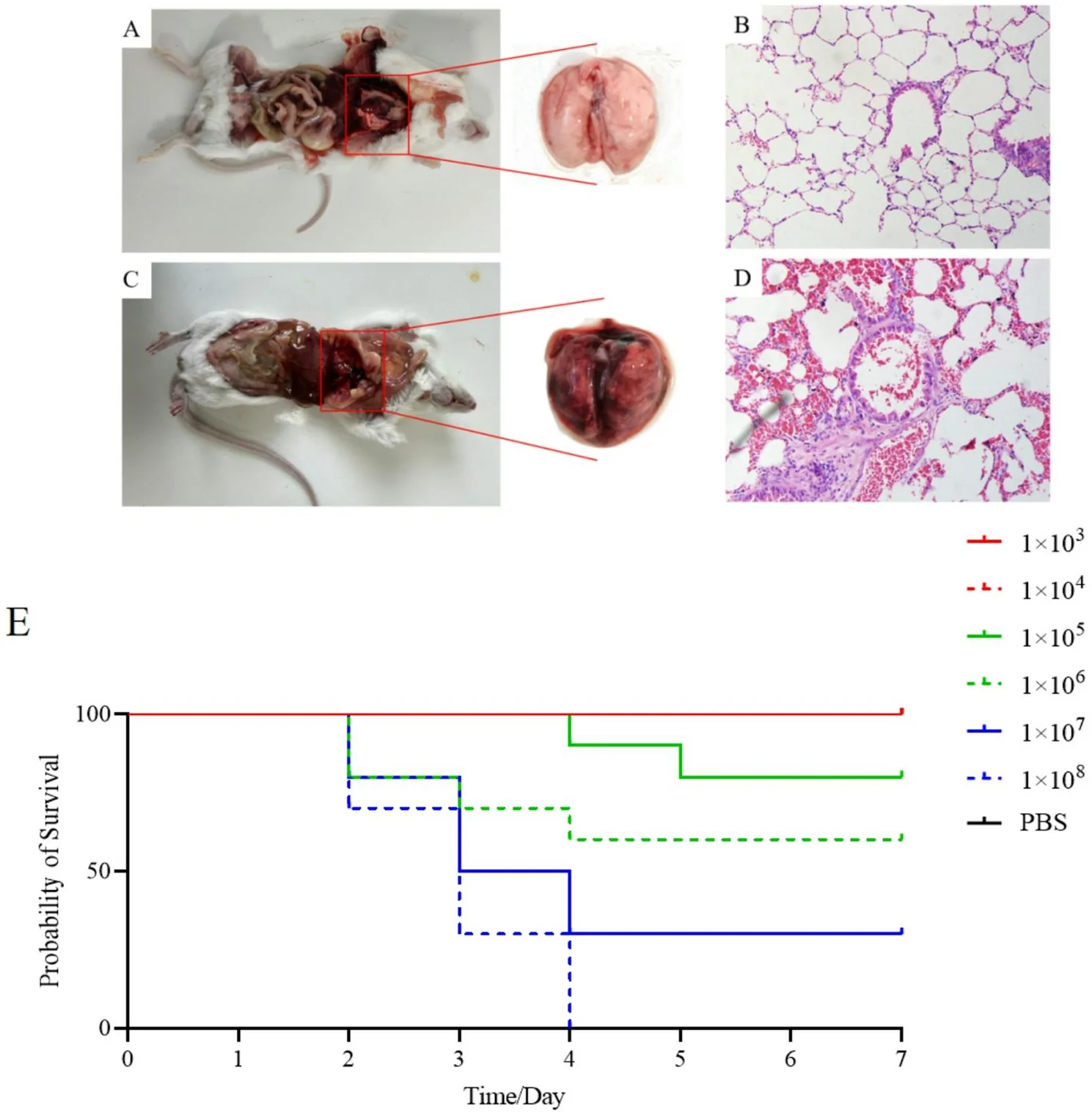
Gross autopsy, pulmonary histopathology and survival curve analysis of Kunming mice after intranasal *E. coli* challenge. **A** Necropsy view of PBS mock-infected control mice, with inset showing intact normal lung tissue; **B**. H&E-stained lung sections of PBS group (200× magnification), presenting normal alveolar morphology; **C**. Necropsy view of E. coli-infected mice, inset showing severe lung hemorrhage and congestion; **D**. H&E-stained lung sections of infected mice (200× magnification), exhibiting extensive alveolar hemorrhage and interstitial inflammatory infiltration; **E**. Kaplan-Meier survival curves of mice administered gradient E. coli inocula (1 × 10³ to 1 × 10⁸ CFU) or PBS control within a 7-day observation period for LD₅₀ calculation

Five strains of *E. coli* isolated from different regions were randomly selected for intranasal infection of Kunming mice at a dose of 2 × 10^6^ CFU per mouse. After challenge, bacteria-infected mice in all groups exhibited marked lethargy and began dying at 48 hpi, whereas no deaths occurred in the PBS group. Kaplan-Meier survival curves are shown in Fig. S1. Necropsy of the deceased mice revealed marked hemorrhage in the lungs, and *E. coli* was successfully isolated from the lungs collected from mouse carcases of the deceased mice (Fig. 5CD). These results confirm the virulence potential of the isolates under artificial experimental conditions in mice. Using the mouse model, we further evaluated the pathogenicity of one of the *E. coli* isolates. Results showed that the LD_50_ of the isolate in mice was 1.58 × 10^6^ CFU (95% confidence interval: 1.23 × 10⁶ to 2.03 × 10⁶ CFU) (Fig. 5E).

### The *E. coli* isolates carry multiple virulence associated genes

**Fig. 6:**
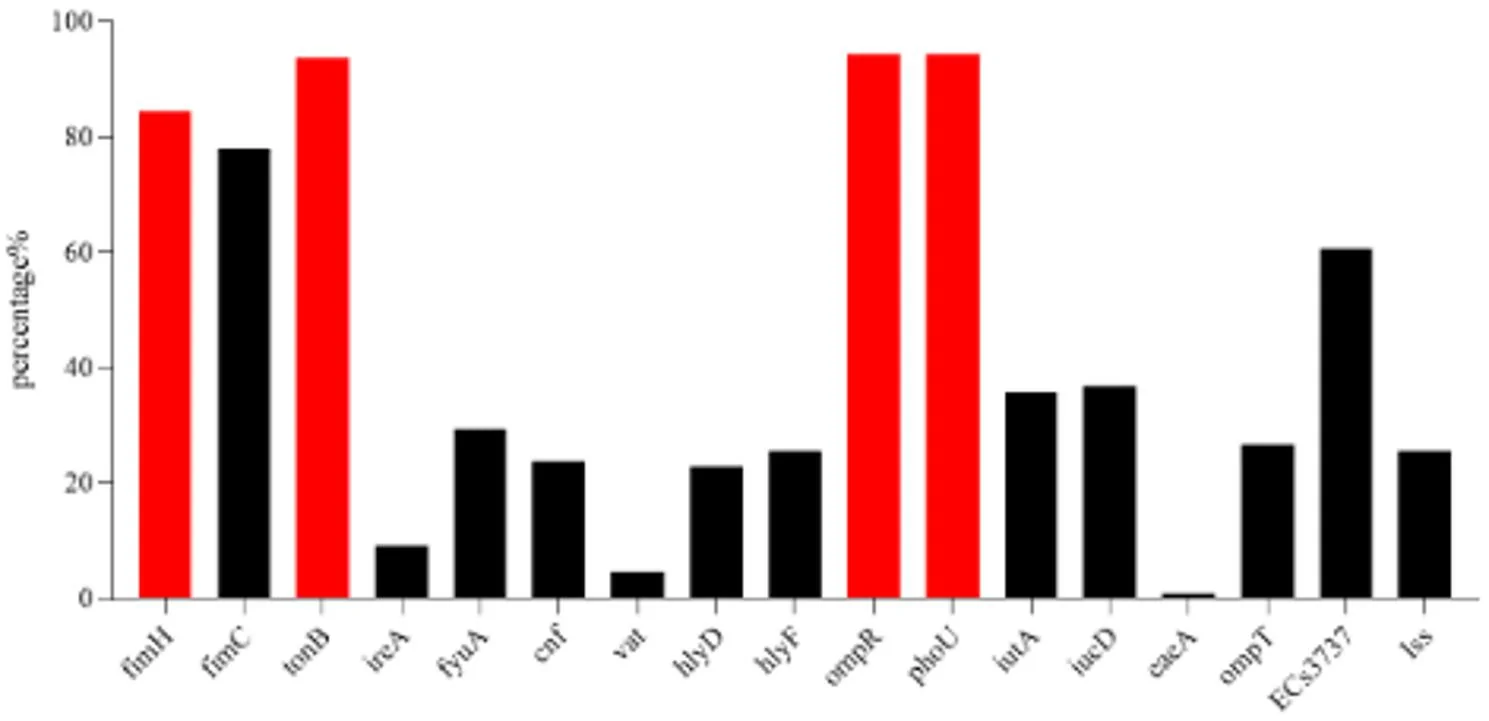
Positive detection rates of functionally classified virulence genes from 109 *E. coli* isolates recovered from pneumonic farmed raccoon dogs. Columns indicate the positive percentage of each tested virulence gene, which were categorized into adhesion, iron uptake, toxin, immune evasion and pathogenicity island-associated functional groups

Virulence-associated gene detection was performed by PCR as described in the Materials and Methods. The most prevalent genes were *ompR* (94.5%), phoU (94.5%), *tonB* (93.58%), and *fimH* (84.4%) (Fig. 6). The high prevalence of these genes suggests they may play important roles in the pathogenicity of raccoon dog *E. coli* isolates, however, functional studies are needed to confirm their actual contribution. Genes were grouped into functional clusters including adhesion, iron acquisition, and virulence regulation. The ETT2 pathogenicity island was detected in 76.1% of isolates; however, this study did not assess whether the island was intact or functional.

### The *E. coli* isolates display severe multidrug resistance

**Fig. 7:**
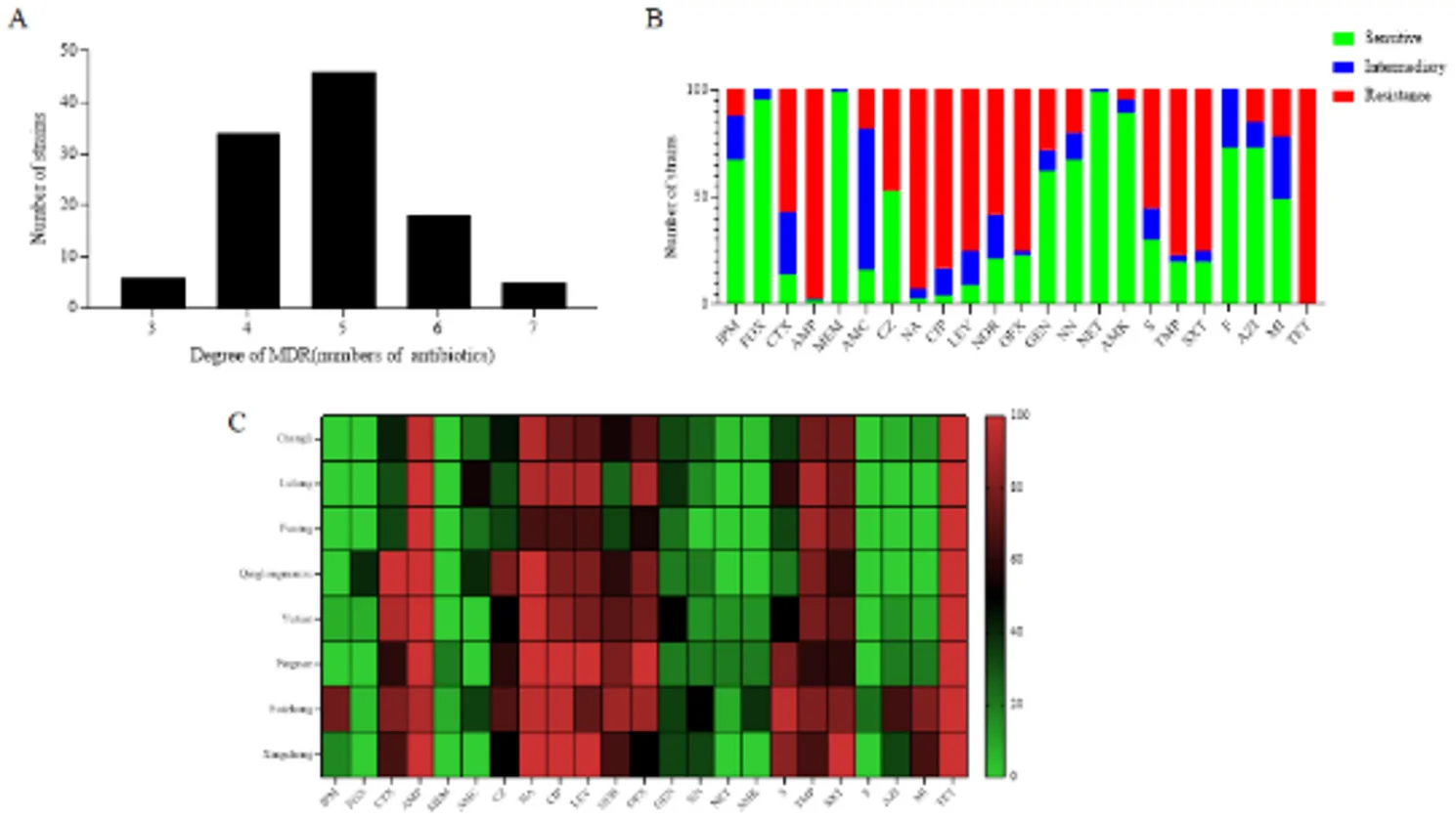
Statistical analysis of antibiotic resistance in *E. coli* isolates. **A** Statistics of multidrug-resistant strains. **B** Resistance rates of *E. coli* isolates to 23 antimicrobial agents IPM Imipenem, F Nitrofurantoin, NA Nalidixic acid, TMP Trimethoprim, FOX Cefoxitin, CTX Cefotaxime, CIP Ciprofloxacin, SXT Sulfamethoxazole, AZI Azithromycin, GEN Gentamicin, AMP Ampicillin, MEM Meropenem, NN Tobramycin, NET Netilmicin, LEV Levofloxacin, MI Minocycline, AMK Amikacin, S Streptomycin, AMC Amoxicillin, NOR Norfloxacin, OFX Ofloxacin, TET Tetracycline, CZ Cephalothin. **C** Antibiotic resistance statistics across different regions

Antimicrobial susceptibility testing revealed a high prevalence of multidrug resistance among the *E. coli* isolates. All isolates were resistant to at least three antimicrobial classes, with a 100% MDR rate (Fig. 7A). For each specific antibiotic, the highest resistance rates were observed for tetracycline (100%), ampicillin (97.25%), nalidixic acid (93.58%), and ciprofloxacin (84.4%), while lower resistance rates were observed for cefoxitin (3.67%), meropenem (2.75%), and netilmicin (5.5%) based on CLSI M100-S32 (Fig. 7B).

There were significant differences in the resistance profiles of isolates from different regions, with strains from Suizhong county of Liaoning and Qinglong county of Hebei, exhibiting overall higher resistance levels (Fig. 7C).

Additionally, some isolates exhibited non-susceptibility to carbapenem antibiotics, with an imipenem resistance rate of 19.27% (21/109) by initial disk diffusion testing, which was further verified by MIC assay as described in the methods section, warranting attention.

Quinolone resistance-associated genes were widely detected across the isolates. Among them, quinolone-resistance-associated chromosomal target loci *gyrA*, *gyrB* and *parC* were detected at rate of 93.58%, 94.5% and 93.58%, respectively, and the plasmid-mediated quinolone resistance *oqxB* exhibited a prevalence of 68%. Integron-associated resistance genes followed with an overall detection rate of 82.57%. The detection rates of peptide, sulfonamide, and aminoglycoside resistance genes were 62.39%, 57.8%, and 48.62%, respectively. And the detection rates of β-lactam, amphenicol, macrolide, and tetracycline resistance genes ranged from 3% to 30% (Table 3).

**Table 3:** Antimicrobial resistance gene test results

Classes of resistance genes	Drug resistant genes name	The number of positives/ total samples	Detection rate
β-lactam	*bla*_*TEM*_	0/109	0
	*bla*_*SHV*_	0/109	0
	*bla*_*OXA*_	0/109	0
	*bla*_*CTX−M*_	26/109	23.85
	*bla*_*CMY*_	6/109	5.5
Quinolone-resistance-associated chromosomal target loci	*gyrA*	102/109	93.58
	*gyrB*	103/109	94.5
	*parC*	102/109	93.58
Plasmid-mediated quinolone resistance (PMQR) genes	*oqxB*	75/109	68.81
	*qnrS*	0/109	0
Aminoglycoside	*aac(3’)-iia*	43/109	39.45
	*aad A1*	53/109	48.62
	*aad B*	0/109	0
Amphenicols	*floR*	44/109	40.37
	*fexA*	2/109	1.83
	*pexA*	0/109	0
Macrolide	*mph(A)*	4/109	3.67
Tetracycline	*tetA*	0/109	0
	*tetB*	26/109	23.85
	*tetC*	0/109	0
Sulfonamides	*sul1*	36/109	33.03
	*sul2*	63/109	57.8
Peptides	*mcr-1*	68/109	62.39
Integrons	*intl1*	90/109	82.57
	*intl2*	0	0

## Discussion

Bacterial pneumonia severely restricts fur animal farming due to high mortality and economic losses. Distinct dominant pneumonia pathogens have been documented in minks and foxes, yet the primary pathogen linked to raccoon dog pneumonia remains unclear. Currently, *Pseudomonas aeruginosa* and *Escherichia coli* have been reported as the dominant pathogens causing pneumonia in farmed minks and foxes, respectively [16]. However, in farmed raccoon dogs, although *E. coli*, *Klebsiella pneumoniae*, *P. aeruginosa* and other bacteria have been reported to be isolated from pneumonia cases, the confirmed primary causative agent of the disease remains unclear. In this study, 159 pneumonia samples were collected from intensive raccoon dog farming areas in eastern Hebei and southern Liaoning provinces of China for bacterial isolation and identification. This study identified *E. coli* as the most frequently isolated bacterium in farmed raccoon dogs in these areas. Further comprehensive evaluation of the pathogenicity and antimicrobial resistance characteristics of these isolates revealed that the isolates displayed notable pathogenic potential in experimental models, high prevalence of MDR, multiple key virulence genes, and a highly complex resistance gene profile.

Multiple pathogens, including bacteria and viruses, are responsible for pneumonia in fur-bearing animals. Compared with viral pneumonia, bacterial pneumonia is characterized by rapid onset and short clinical course, often resulting in death within a few days after the appearance of clinical signs. Previous studies have reported that *Pseudomonas aeruginosa* is the dominant causative agent of hemorrhagic pneumonia in minks, while *E. coli* is the primary pathogen responsible for pneumonia in foxes [17]. In the present study, *E. coli* was the most frequently isolated bacterium associated with bacterial pneumonia in farmed raccoon dogs, while isolation rate alone cannot confirm definite etiological causality, and further validation including healthy control groups, viral pathogen screening and in vivo challenge on raccoon dogs is required in future work.

*Escherichia coli* can establish multiple types of infection in a variety of fur-bearing animal species, and the pneumonia caused by this pathogen is particularly fatal [18]. In this study, we found that although *E. coli* infection in raccoon dogs predominantly manifested as clinical signs of pneumonia, the detection of bacteria and pathological lesions in diverse visceral organs is suggestive of systemic bacterial dissemination across multiple organs, which is consistent with possible systemic dissemination of the bacterium. However, definitive evidence that *E. coli* directly caused multi-organ lesions is still lacking, as quantitative bacterial load and strain-level identity across organs were not verified in this study. Large quantities of *E. coli* were successfully isolated from the lung, liver, spleen, kidney, blood, and brain of diseased raccoon dogs, and severe histopathological lesions were observed in these affected organs. Notably, quantitative bacterial load measurements, strain homology verification across different organs, and comprehensive sterility control records were not generated in this study; therefore, these culture results cannot serve as definitive proof of systemic *E. coli* infection.

The fecal-oral route is recognized as the classic transmission route of *E. coli*. However, this may not be the dominant transmission pattern in fur-bearing animals. We observed that the outbreak of pneumonia in fur-bearing animals in China mainly occurs from October to November, when the weather turns cold and the animals undergo molting. This hypothesis was further supported by our experimental infection assay in mice: for the same bacterial strain, intranasal inoculation, compared with oral administration, was much more efficient in inducing pneumonia and causing death in mice. Additionally, factors such as weakened immunity caused by the cold weather and molting in the fall, windy conditions that facilitate the spread of bacteria-carrying vectors, and the inhalation of bacteria through coughing of sick animals may also contribute to the outbreak of bacterial pneumonia. Of course, this requires further evaluation through experimentation.

Serotyping is an important tool for the development of *E. coli* inactivated vaccines but is not the sole basis [19]. In this study, a total of 6 serotypes were identified from 109 *E. coli* isolates, among which serotypes O8 (55.05%), O25 (11.01%), and O78 (16.51%) were the predominant serotypes. It is well established that O78 is a classic dominant serotype of avian pathogenic *E. coli* (APEC), while O25 is a globally prevalent multidrug-resistant serotype commonly recovered from human and animal extraintestinal infections. Both serotypes have been sporadically reported in mink-derived pathogenic *E. coli*. In contrast, serotype O8 was identified as the absolute dominant serotype in this study, which has rarely been reported as the major epidemic serotype of *E. coli* from fur-bearing animals previously [20, 21]. This result suggests possible that the epidemic serotypes of raccoon dog-derived *E. coli* exhibit significant geographical and host specificity, which is the core reason for the limited protective efficacy of existing commercial *E. coli* vaccines in raccoon dog farming. Of course, performing only the O-serotyping is limited, and additional studies including H-antigen typing and whole-genome sequencing will be performed in our following work.

The ability to evade persistent clearance by the host immune system is a prerequisite for respiratory bacterial pathogens to induce pneumonia and even septicemia [22]. Histopathological examination of the tissues and organs from diseased raccoon dogs revealed consistent aggregation and infiltration of inflammatory cells. To elucidate the molecular basis of the pathogenicity of *E. coli* isolates from raccoon dogs with pneumonia, virulence gene profiling was performed on all isolates in this study. All isolates harbored at least 5 virulence genes, among which the immune evasion-related genes *ompR* and *phoU* were detected at an extremely high rate of 94.5%, the iron acquisition-related gene *tonB* at 93.58%, and the adhesion and colonization-related genes *fimH* and *fimC* at 84.4% and 77.98%, respectively. High carriage rates of *fimH*, *fimC*, and *ompR* have been reported in *E. coli* strains causing pneumonia in various fur-bearing animals, chikens and canines [8, 23, 24]. In this study, we found that the high prevalence of these genes further supports the crucial role of these virulence factors in *E. coli*-induced pneumonia. The type 1 fimbriae encoded by *fimH* and *fimC* are the key structures mediating the adhesion of *E. coli* to the respiratory epithelial mucosa, which is the initial step for bacterial colonization in the respiratory tract and subsequent pneumonia development. The *tonB* gene is the core regulatory factor of the bacterial iron acquisition system, which facilitates bacterial iron acquisition in the nutrient-restricted host environment, thereby enhancing bacterial survival and proliferation in vivo. The *ompR* and *phoU* genes are involved in the regulation of bacterial outer membrane protein expression and environmental adaptation, which help bacteria escape host immune clearance. Notably, the ETT2 pathogenicity island-associated gene *Ecs3737* was detected in 60.55% of the isolates. This gene has been confirmed to be closely related to the pathogenicity of APEC, but its high prevalence in raccoon dog-derived *E. coli* has not been reported previously [25]. The coexistence of multiple virulence genes provides a potential molecular explanation for the robust pathogenicity observed in artificial mouse intranasal challenge assays, yet direct causal linkage between these virulence genotypes and spontaneous pneumonia in raccoon dogs remains unconfirmed due to the absence of in vivo challenge experiments in the natural host.

Antibiotics are widely used to treat bacterial pneumonia in farmed fur animals. However, due to the inappropriate use of antibiotics, animal-derived *E. coli* have shown a growing trend of severe antimicrobial resistance [26]. Previous studies have demonstrated that multiple antimicrobial-resistant *E. coli* strains exist in wild raccoon dogs, and these strains possess strong biofilm formation ability, which promotes bacterial survival and transmission in the environment [27]. In this study, samples were collected from farmed raccoon dogs, and we similarly found that pneumonia-causing *E. coli* isolates exhibited a high rate of multidrug resistance (MDR), especially significant resistance to commonly used antibiotics Meanwhile, significant differences in the antimicrobial resistance profiles of *E. coli* isolates from different regions were observed in this study, which may be related to the differences in medication practices and breeding management levels among different regions. This finding suggests that clinical medication regimens should be adjusted based on local antimicrobial resistance surveillance data to ensure precise treatment and slow the evolution of antimicrobial resistance.

Antimicrobial resistance gene detection provides preliminary insights into the genetic basis underlying the high prevalence of resistance phenotypes of the isolates. The detection rates of quinolone resistance-associated chromosomal target genes *gyrA*, *gyrB*, and *parC* all exceeded 93%, which was consistent with the high resistance rate of the isolates to quinolone antibiotics. It is noteworthy that the integron gene *int1* was detected in 82.57% of the isolates. Integrons are important mobile genetic elements (MGEs) that can capture and disseminate antimicrobial resistance genes among bacteria, and are the core driving force for the development and transmission of MDR in Gram-negative bacteria. Its high prevalence explains the widespread coexistence of multiple resistance genes in the isolates.

The detection of *mcr-1* and carbapenem non-susceptibility in raccoon dog-derived *E. coli* raises concerns regarding potential public health implications, as these isolates may serve as a reservoir of clinically relevant resistance genes. However, the actual transmission risk to humans and the environment via direct contact, farming wastewater, fur processing or other routes remains unconfirmed, and requires further multi-source epidemiological investigation including environmental sampling, plasmid characterization, conjugation transfer assays and human exposure assessment.

Concerning the complex pattern of antibiotic resistance gene carriage in raccoon dog-derived *E. coli*, further epidemiological studies are essential, including whole-genome sequencing, plasmid typing, and human exposure assessments.

There are still some limitations in this study that need to be addressed in subsequent research. First, the samples in this study were only collected from eastern Hebei Province and southern Liaoning Province, so the results cannot fully represent the epidemic characteristics of *E. coli* pneumonia in raccoon dogs nationwide. A nationwide epidemiological survey is required in the future to clarify the national epidemic pattern of pathogenic *E. coli* from raccoon dogs. Second, this study lacks a healthy control group for comparison, viral pathogen screening, quantitative bacterial load analysis, and strain-level molecular typing of isolates from different organs, which limits the definitive confirmation of *E. coli* as the causative agent of pneumonia and systemic lesions. Third, only O-antigen serotyping was performed on the isolates in this study, while H-antigen typing and whole-genome sequencing (WGS) analysis were not conducted, which limits the in-depth investigation of the phylogenetic evolution, serotype diversity, and molecular transmission mechanism of the strains. Forth, the pathogenicity verification was only completed in a mouse model in this study, and subsequent in vivo challenge tests in raccoon dogs are needed to further verify the pathogenicity of the isolates in their natural host. Fifth, this study only detected the carriage of antimicrobial resistance genes, but did not verify the expression level of resistance genes and the horizontal transmission ability of MGEs, and the related mechanisms need to be further elucidated in follow-up studies. Finally, carbapenemase-encoding genes were not screened in this study, and the underlying mechanism of carbapenem non-susceptibility remains to be clarified.

## Conclusions

In conclusion, this study identified *E. coli* as the most frequently isolated bacterium associated with bacterial pneumonia in farmed raccoon dogs in eastern Hebei and southern Liaoning provinces, China. The isolates exhibited high virulence potential in mouse model, carried multiple virulence genes, and showed a high prevalence of multidrug resistance. O8, O25, and O78 were the most frequently detected O-serotypes. These findings provide important baseline data for understanding the epidemiology of *E. coli*-associated pneumonia in raccoon dogs and guiding clinical antimicrobial use. The presence of *mcr*-1 and potential carbapenem non-susceptibility suggests that these isolates may represent a reservoir of clinically relevant resistance genes that warrants further surveillance. Further studies are needed to confirm the etiological role of *E. coli* in raccoon dog pneumonia, evaluate vaccine candidates, and assess the actual public health risk posed by these isolates.

## Supplementary Information

Supplementary Material 1.

Supplementary Material 2.

Supplementary Material 3.

## Data Availability

The original contributions presented in the study are included in the article material, further inquiries can be directed to the corresponding author.
