# Supplementary material for: High prevalence of virulent and multidrug-resistant Escherichia coli as the most frequently isolated bacterium associated with bacterial pneumonia in farmed raccoon dogs
Source: BMC Vet Res. 2026 Jul 31;22:636. doi: 10.1186/s12917-026-05773-5 (PMC13715278; doi:10.1186/s12917-026-05773-5)
Supplement: Supplementary file 1 [file 12917_2026_5773_MOESM1_ESM.pdf]

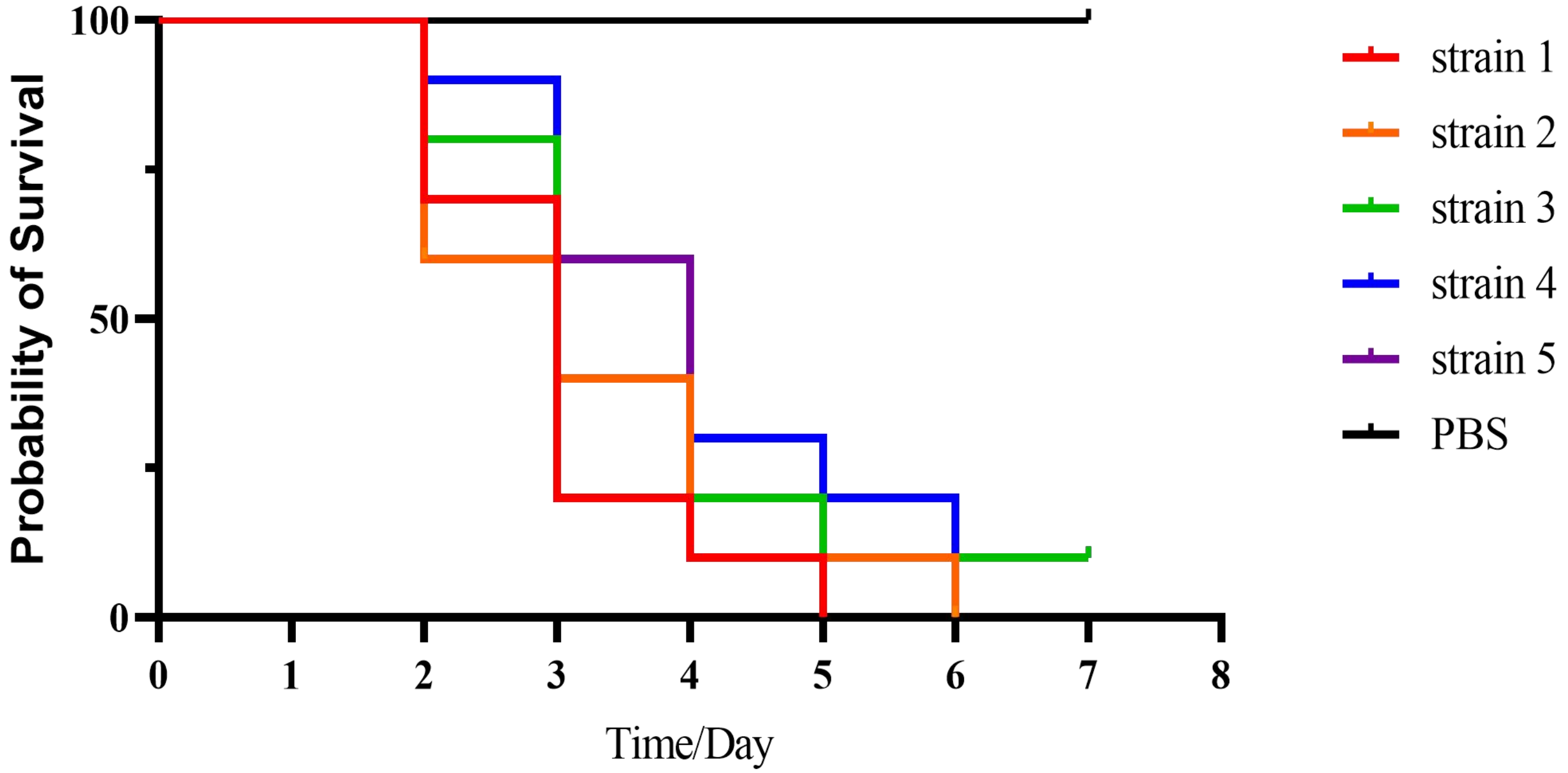

Figure S1. Kaplan-Meier survival curves of Kunming mice infected with five E. coli strains (2×10^6 CFU per mouse) via intranasal administration. PBS group was the uninfected control. All infected groups exhibited lethargy and mortality starting at 48 hpi (2d), with zero death in PBS group during observation.
