# Supplementary material for: High prevalence of virulent and multidrug-resistant Escherichia coli as the most frequently isolated bacterium associated with bacterial pneumonia in farmed raccoon dogs
Source: BMC Vet Res. 2026 Jul 31;22:636. doi: 10.1186/s12917-026-05773-5 (PMC13715278; doi:10.1186/s12917-026-05773-5)
Supplement: Supplementary file 2 [file 12917_2026_5773_MOESM2_ESM.docx]

Supplementary Table 1 Strain Info

| **Strains** | **Serotype** | **Drug-resistant gene** | **Virulence gene** | **Location** |
| --- | --- | --- | --- | --- |
| Strain 1 | O1 | *gyrA, gyrB, parC, sul1, tetB* | *cnf, Ecs3737, fimC, fimH, fyuA, hlyD, hlyF, ireA, Iss, iucD, iutA, ompR, ompT, phoU, tonB* | Huludao City, Liaoning Province |
| Strain 2 | O25 | *aac(3')-iia, aadA1, gyrA, gyrB, mcr-1, parC* | *cnf, fimC, fimH, fyuA, hlyD, iucD, iutA, ompR, phoU, tonB* | Huludao City, Liaoning Province |
| Strain 3 | O78 | *aadA1, gyrA , gyrB, parC, sul1, sul2* | *Ecs3737, fimC, fimH, fyuA, hlyF, Iss, iucD, iutA, ompR, ompT, phoU, tonB* | Qinhuangdao City, Hebei Province |
| Strain 4 | O8 | *gyrA, gyrB, int1, mcr-2, oqxB, parC, sul2, tetB* | *cnf, Ecs3737, hlyD, hlyF, Iss, iucD, iutA, ompR, ompT, phoU, TonB* | Qinhuangdao City, Hebei Province |
| Strain 5 | O18 | *aac(3')-iia, aadA1, floR, gyrA, gyrB, int1, mcr-1, oqxB, parC, sul1, sul2, tetB* | *Ecs3737, fimC, fimH, fyuA, ireA, ompR, phoU, tonB* | Tangshan, Hebei Province |
