# Supplementary material for: High prevalence of virulent and multidrug-resistant Escherichia coli as the most frequently isolated bacterium associated with bacterial pneumonia in farmed raccoon dogs
Source: BMC Vet Res. 2026 Jul 31;22:636. doi: 10.1186/s12917-026-05773-5 (PMC13715278; doi:10.1186/s12917-026-05773-5)
Supplement: Supplementary file 3 [file 12917_2026_5773_MOESM3_ESM.docx]

Supplement Table 2 MIC method was used to recheck drug-resistant strains for meropenem and imipenem

| **Strains** | **Imipenem MIC(μg/mL)** | **Strains** | **Imipenem MIC(μg/mL)** |
| --- | --- | --- | --- |
| 70 | 32 | 91 | 16 |
| 78 | 8 | 92 | 8 |
| 79 | 32 | 93 | 64 |
| 81 | 4 | 95 | 32 |
| 82 | 8 | 96 | 4 |
| 83 | 8 | 98 | 64 |
| 84 | 4 | 99 | 32 |
| 87 | 8 | 101 | 64 |
| 88 | 16 | 103 | 4 |
| 89 | 4 | 107 | 16 |
| 90 | 64 |  |  |
|  |  |  |  |
| **Strains** | **Meropenem MIC(μg/mL)** |  |  |
| 76 | 8 |  |  |
| 94 | 16 |  |  |
| 99 | 32 |  |  |
|  |  |  |  |

Note: According to CLSI standards, the susceptibility breakpoint for imipenem and meropenem is ≤1 µg/ml, intermediate is 2 µg/ml, and resistant is ≥4 µg/ml.
